# Ecological Momentary Assessment and Machine Learning for Predicting Suicidal Ideation Among Sexual and Gender Minority Individuals

**DOI:** 10.1001/jamanetworkopen.2023.33164

**Published:** 2023-09-11

**Authors:** Chang Lei, Diyang Qu, Kunxu Liu, Runsen Chen

**Affiliations:** 1 Vanke School of Public Health, Tsinghua University, Beijing, China; 2 Institute for Healthy China, Tsinghua University, Beijing, China

## Abstract

**Importance:**

Suicidality poses a serious global health concern, particularly in the sexual and gender minority population. While various studies have focused on investigating chronic stressors, the precise prediction effect of daily experiences on suicide ideation remains uncertain.

**Objective:**

To test the extent to which mood fluctuations and contextual stressful events experienced by sexual and gender minority individuals may predict later short- and long-term suicide ideation.

**Design, Setting, and Participants:**

This diagnostic study collected twice-daily data on mood states and stressful events from sexual and gender minority individuals over 25 days throughout 3 waves of the Chinese Lunar New Year (before, during, and after), and follow-up surveys assessing suicidal ideation were conducted 1, 3, and 8 months later. Online recruitment advertisements were used to recruit young adults throughout China. Eligible participants were self-identified as sexual and gender minority individuals aged 18 to 29 years. Those who were diagnosed with psychotic disorders (eg, schizophrenia spectrum or schizotypal disorder) or prevented from objective factors (ie, not having a phone or having an irregular sleep rhythm) were excluded. Data were collected from January to October 2022.

**Main Outcomes and Measures:**

To predict short-term (1 month) and longer-term (3 and 8 months) suicidal ideation, the study tested several approaches by using machine learning including chronic stress baseline data (baseline approach), dynamic patterns of mood states and stressful events (ecological momentary assessment [EMA] approach), and a combination of baseline data and dynamic patterns (EMA plus baseline approach).

**Results:**

A total of 103 sexual and gender minority individuals participated in the study (mean [SD] age, 24.2 [2.5] years; 72 [70%] female). Of these, 19 (18.4%; 95% CI, 10.9%-25.9%), 25 (24.8%; 95% CI, 16.4%-33.2%), 30 (29.4%; 95% CI, 20.6%-38.2%), and 32 (31.1%; 95% CI, 22.2%-40.0%) reported suicidal ideation at baseline, 1, 3, and 8 months follow-up, respectively. The EMA approach showed better performance than the baseline and baseline plus EMA approaches at 1-month follow-up (area under the receiver operating characteristic curve [AUC], 0.80; 95% CI, 0.78-0.81) and slightly better performance on the prediction of suicidal ideation at 3 and 8 months’ follow-up. In addition, the best approach predicting suicidal ideation was obtained during Lunar New Year period at 1-month follow-up, which had a mean AUC of 0.77 (95% CI, 0.74-0.79) and better performance at 3 and 8 months’ follow-up (AUC, 0.74; 95% CI, 0.72-0.76 and AUC, 0.72; 95% CI, 0.69-0.74, respectively).

**Conclusions and Relevance:**

The findings in this study emphasize the importance of contextual risk factors experienced by sexual and gender minority individuals at different stages. The use of machine learning may facilitate the identification of individuals who are at risk and aid in the development of personalized process-based early prevention programs to mitigate future suicide risk.

## Key Points

**Question:**

To what extent does the analysis of daily data encompassing mood fluctuations and contextual stressful events effectively predict short- and long-term suicidal ideation in sexual and gender minority individuals?

**Findings:**

This diagnostic study of 103 individuals aged 18 to 29 years found that using 25-day ecological momentary assessment yielded acceptable prediction performance on 1-, 3-, and 8-month suicidal ideation. The prediction effect of feelings faded over time, while the prediction effect of contextual events remained strong.

**Meaning:**

The findings suggest a promising future for detecting suicide ideation over time through the analysis of data on specific types of mood fluctuations and contextual events within a few days.

## Teaser

This diagnostic study uses machine learning to evaluate moods and stressful events preceding suicidal ideation among sexual and gender minority individuals in China.

## Introduction

Sexual and gender minority is an umbrella term that includes individual who identify as lesbian, gay, bisexual, transgender, 2-spirit, queer, and intersex, among others. Compared with heterosexual and cisgender individuals, sexual and gender minority populations are at a higher risk of experiencing mental health problems.^1^ For instance, a national-level survey conducted in China revealed discrimination experienced by Chinese sexual and gender minority groups in their daily lives.^2,3^

Suicide prevention and intervention are essential supports for sexual and gender minority individuals. Previous studies in this area have predominantly focused on chronic stressors. For example, the minority stress model^4^ suggests that sexual minority individuals encounter hostile stressors that can have detrimental effects on their health.^5,6^ This model may neglect the impact of dynamic interactions between mood fluctuations in sexual and gender minority populations triggered by stressful life events, creating barriers to developing early-stage interventions to reduce risk.^4,7^ Following the fluid vulnerability theory of suicide, which conceptualizes risk as a nonlinear inherently dynamic construct, single measures of chronic stress levels or stable mental health symptoms have not been shown to produce more accurate predictions of short-term or longer-term suicidal ideation in this population.^8,9^

Additionally, it is important to take an individual’s cultural background into consideration. The Lunar New Year is a unique holiday for Chinese people, lasting for 16 days, during which extended family members gather together for celebration.^10^ For some sexual and gender minority individuals, the increased frequency of family gatherings during the period can further exacerbate conflicts with family members,^11^ given young people are often asked about marriage and children.^12^ This issue is particularly important in Chinese culture, where Confucian influence supports the belief that “the worst forms of unfilial conduct are to have no descendants.”^13^

Hence, it is crucial to use more precise and immediate time-interval measures that specifically take cultural backgrounds into account, such as self-report from ecological momentary assessment (EMA).^14^ The integration of EMA and machine learning further offers potential for identifying risk patterns and making predictions over time.^15^ This in turn assists practitioners in personalizing interventions by determining the most effective strategies at different time points.

However, to our knowledge, no studies have investigated the extent to which mood fluctuations and stressful events experienced by sexual and gender minority individuals during Chinese Lunar New Year may predict later suicide ideation. In this study, we used machine learning to predict short-term (1 month) and longer-term suicidal ideation (3 months and 8 months) using the following input: baseline data, dynamic patterns of mood states and stressful events, and a combination of baseline data and dynamic patterns.

## Method

### Participants and Procedures

Eligible participants were aged between 18 and 29 years, self-identified as sexual and gender minority, and resided in China. Participants who were diagnosed with psychotic disorders (eg, schizophrenia spectrum, schizotypal disorder) or prevented from objective factors (ie, not having a phone or having an irregular sleep rhythm) were excluded.

Internet-based recruitment advertisements were published. After signing digital consent forms, participants completed baseline assessment. They received daily EMA surveys by messages at 9:50 am and 9:00 pm for 25 days and had 2 hours to complete the survey. Each initial message was followed by a 1-hour reminder. Follow-up took place at 1, 3, and 8 months after the completion of EMA. Participants received compensation proportional to response rates, with a base fee of 100 CNY awarded to those who completed at least 60% of daily surveys. An additional 100 CNY was received after completing follow-up. The study’s protocol was authorized by Tsinghua University institutional review board. Our study followed the Transparent Reporting of a Multivariable Prediction Model for Individual Prognosis or Diagnosis (TRIPOD) reporting guideline.

### Measures

#### Baseline Assessment

Demographic information collected participants’ age, sex at birth, sexual orientation, gender identity, ethnicity, educational level, occupation, marital status, and average monthly expenditure. The baseline assessment also included measurements to assess participants’ baseline suicidal ideation, lifetime nonsuicidal self-injury frequency, lifetime suicide attempt history, psychiatric diagnosis history, psychiatric treatment history, family history of suicide attempt, and family history of psychiatric diagnosis.

#### Daily Emotional Mental State

Six modified items from Multidimensional Mood Questionnaire were used to measure emotional mental states.^16^ Participants recorded current emotions with “At this moment, I feel”: (1) tired-awake; (2) content-discontent; (3) agitated-calm; (4) full of energy-without energy; (5) ill-well; (6) relaxed-tense. End point scores ranged from 0 to 6.

#### Daily Negative Stressful Event

Three items were measured using the heterosexist harassment, rejection, and discrimination scale^17^: (1) “Have you experienced any of the following negative stresses due to your sexual and gender minority identification since the last prompt?” (2) “Which of these stresses has had the most impact on you?” and (3) “How much does it worry you?” The sources of stress for items 1 and 2 included marriage, fertility, economy, housing, medical care, family discrimination, working, learning, socializing, and other. Participants could access item 3 if they chose any option other than none.

#### Suicidal Ideation

The final item of the 9-item Patient Health Questionnaire (PHQ-9),^18^ “thoughts that you would be better off dead, or of hurting yourself,” was used to determine the frequency of suicidal ideation.^19,20,21^ This item was dichotomized based on the presence (score of 1 or above) or absence (score of 0) of any suicidal ideation; thus, a score of 1 indicated the presence of suicidal ideation occurring on at least several days.

### Statistical Analysis

All analyses were done using Python version 3.9 (Python Software Foundation), Scikit-learn version 1.0.2 (Scikit-learn Contributors), and LightGBM version 3.3.2 (LightGBM Library). Using *t* tests, 2-sided *P* values less than .05 were considered significant.

#### Baseline Predictor Preparation

We used participants’ baseline suicidal ideation, lifetime nonsuicidal self-injury frequency, lifetime suicide attempt history, psychiatric diagnosis history, psychiatric treatment history, family history of suicide attempt, and family history of psychiatric diagnosis as baseline predictors.

#### EMA Predictor Preparation

The EMA data were collected over a 25-day period throughout 3 waves of Chinese Lunar New Year (before, during, and after), observing each individual on 9 variables. Several time-series predictors were extracted from EMA variables. These features included mean, SD, slope, maximum change, and probability of acute change^22^ for daily emotional states and stressful events. For stressful events, we additionally calculated the cumulative person-specific frequency, which is the frequency of specific stressful events and the frequency of specific most stressful events.^23^ A total of 60 features were extracted for each individual in 1 observation.

#### Modeling Approach and Performance

We used lightweight gradient boosted machines (LightGBM) for predicting suicidal ideation^24^ (see the eMethods in Supplement 1 for an exposition of modeling approach). For detailed information on the hyperparameter tuning process, please refer to the eMethods and eTable 1 in Supplement 1.

Five-fold cross-validation with 3 repetitions was used to obtain an estimate of modeling approach performance.^25^ Area under the receiver operating characteristic curve (AUC), sensitivity, specificity, and positive predictive value were used to evaluate performance. Please refer to the eMethods in Supplement 1 for data partition and training process.

#### Predictor Importance

Predictor ranking was computed using a built-in function within LightGBM. LightGBM is a tree-based ensemble model comprising multiple decision-tree models. Consequently, the feature importance can be measured by the frequency of attributes used as split nodes. The greater the utilization of an attribute as a split node, the greater its relative importance. This importance can be explicitly calculated for each feature across an entire data set, enabling ranking and comparison.

#### EMA Missingness and Different Validation Procedures

During the EMA period, data were missing at the survey level rather than at the item level. If a survey was missing, that day’s data were not included.^26^ We did not use imputation, as the configuration of missing data involved a complete survey rather than missing items from a survey.^26^ The modeling approach we used was based on EMA data from Chinese sexual and gender minority individuals with a primary focus on Chinese Lunar New Year. However, this approach has yet to be thoroughly validated in EMA data from other target populations or research contexts. To somewhat alleviate the inadequacy of validations, 2 additional experiments were performed: 10-fold repeated 3 times and leave-one-out cross-validation.

## Results

### Descriptive Characteristics

Of 103 participants (mean [SD] age, 24.2 [2.5] years; 72 [70%] female and 31 [30%] male), 47 individuals were homosexual (45.6%), 27 were bisexual (26.2%), 76 were cisgender (73.8%), and 16 were queer (15.5%). Four individuals exhibited a lifetime nonsuicidal self-injury frequency equal to or greater than 51 occurrences, and most (n = 98) did not report lifetime suicide attempt. For further demographic, diagnostic, and clinical information, please refer to the Table. A flowchart depicting the different steps of study and the number of participants ultimately included is presented in eFigure 1 in Supplement 1. During the EMA period, we obtained a total of 5011 completed surveys with a compliance rate of 94.5%. The presence of suicidal ideation was reported by 19 (18.4%; 95% CI, 10.9%-25.9%), 25 (24.8%; 95% CI, 16.4%-33.2%), 30 (29.4%; 95% CI, 20.6%-38.2%), and 32 (31.1%; 95% CI, 22.2%-40.0%) individuals at baseline, 1, 3, and 8 months’ follow-up, respectively.

**Table.** Demographic Characteristics, Diagnoses, and Clinical Characteristics for 103 Participants

Characteristic	No. (%)
Age, mean (SD), y	24.2 (2.5)
Sex at birth	
Male	31 (30.1)
Female	72 (69.9)
Sexual orientation	
Asexual	2 (1.9)
Homosexual	47 (45.6)
Heterosexual	10 (9.7)
Bisexual	27 (26.2)
Pansexual	14 (13.6)
Unsure	3 (2.9)
Gender identity	
Cisgender	76 (73.8)
Transgender man	6 (5.8)
Transgender woman	4 (3.9)
Queer	16 (15.5)
Unsure	1 (1.0)
Ethnicity	
Han	96 (93.2)
Others	7 (6.8)
Educational level	
High school	2 (1.9)
Bachelor/junior college	83 (80.6)
Master or higher	18 (17.5)
Occupational statement	
Student	77 (74.8)
Employed	21 (20.3)
Unemployed	5 (4.9)
Marriage	
Unmarried	101 (98.1)
Married	1 (1.0)
Divorced	1 (1.0)
Average monthly expenditures, Yuan	
≤2000	42 (40.8)
2001-3000	31 (30.1)
3001-4000	16 (15.5)
4001-5000	6 (5.8)
≥5001	8 (7.8)
Baseline suicidal ideation	
Yes	19 (18.4)
No	84 (81.6)
Lifetime nonsuicidal self-injury frequency	
0	63 (61.2)
1-10	27 (26.2)
11-50	9 (8.7)
≥51	4 (3.9)
Lifetime suicide attempt history	
Yes	5 (4.9)
No	98 (95.1)
Psychiatric diagnosis history	
Generalized anxiety disorder	16 (15.5)
Major depressive disorder	23 (22.3)
Personality disorder	2 (1.9)
Eating disorder	2 (1.9)
Sleep disorder	7 (6.8)
Bipolar disorder	8 (7.8)
Obsessive compulsive disorder	1 (1.0)
Phobia	1 (1.0)
Panic disorder	2 (1.9)
Autism	2 (1.9)
Attention deficit/hyperactivity disorder	2 (1.9)
Other psychiatric disorder	1 (1.0)
Psychiatric treatment history	
Hospitalization	1 (1.0)
Psychiatric medication	12 (11.7)
Psychological counseling/psychotherapy	25 (24.3)
Physical therapy	2 (1.9)
Other treatment	1 (1.0)
Family history of suicide attempt	
Biological father	5 (4.9)
Biological mother	1 (1.0)
Biological siblings	2 (1.9)
Paternal grandfather	1 (1.0)
Other relatives	12 (11.7)
Family history of psychiatric diagnosis	
Biological father	2 (1.9)
Biological siblings	2 (1.9)
Maternal grandmother	2 (1.9)
Paternal grandfather	1 (1.0)
Paternal grandmother	2 (1.9)
Other relatives	10 (9.7)

### Predictive Performance Comparison of Different Modeling Approaches

**Figure 1.**
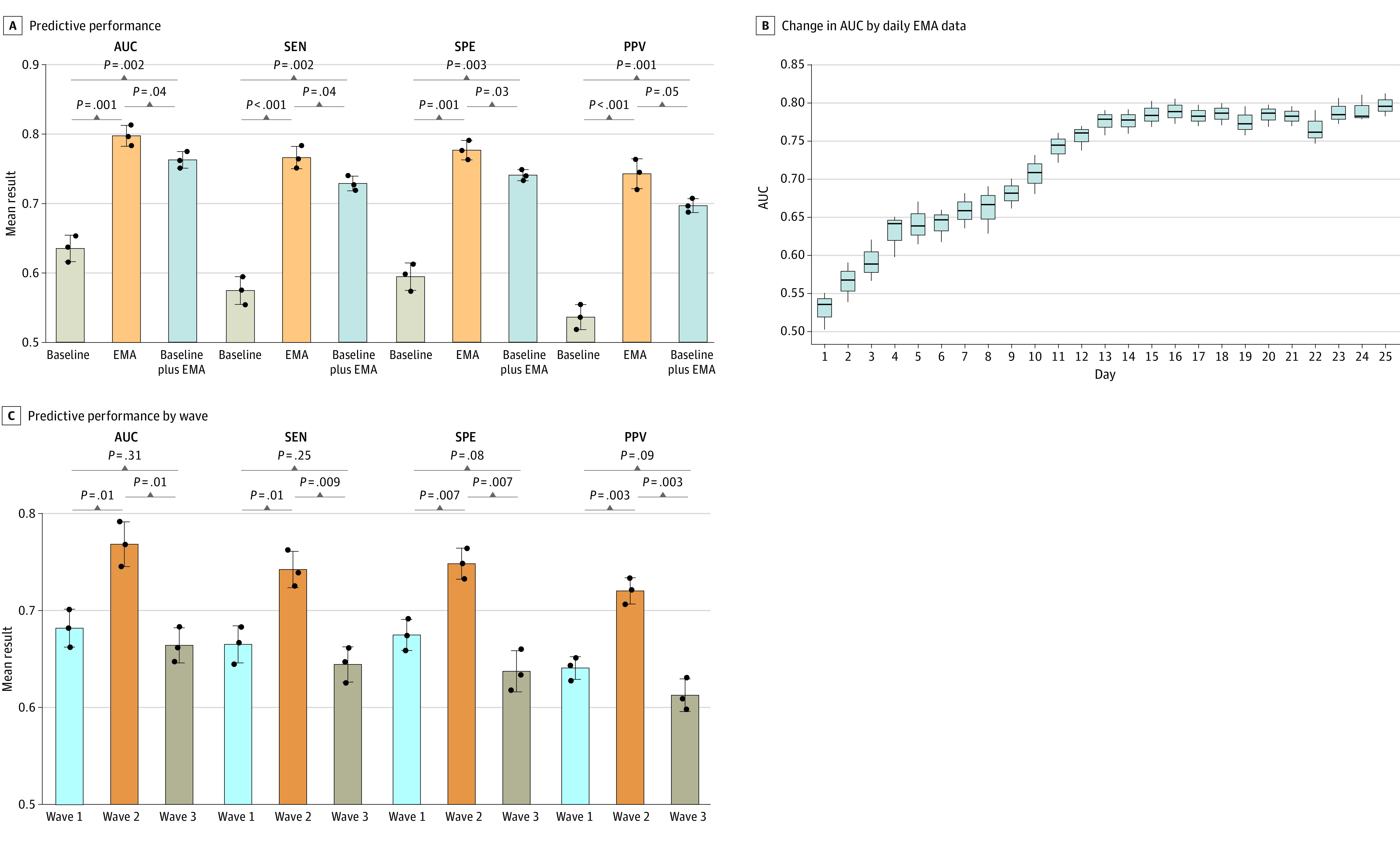
Performance of Modeling Approach in Predicting Suicidal Ideation at 1-Month Follow-Up A, Comparison of predictive performance of using baseline, ecological momentary assessment (EMA), and baseline plus EMA data. B, Changes in the area under the receiver operating characteristic curve (AUC) when the daily EMA data were continually overlaid to predict. C, Evaluation of predictive performance of 3 different waves. Differences between 2 groups were calculated with *t* tests. *P* values were adjusted for multiple comparisons by using false discovery rate adjustment (Benjamini-Hochberg procedure). PPV indicates positive predictive value; SEN, sensitivity; SPE, specificity.

Modeling approach performance metrics for baseline, EMA, and baseline plus EMA at 1, 3, and 8 months’ follow-up are presented in Figure 1 and eFigures 2 and 3 in Supplement 1 (eTable 2 in Supplement 1 has specific values). The EMA approach outperformed the baseline and baseline plus EMA approaches on 4 metrics at 1-month follow-up. Although there was no significant difference in performance metrics when predicting using EMA data and baseline plus EMA data at 3 and 8 months’ follow-up, the average predictive performance obtained using EMA data was better. In particular, the EMA approach achieved the optimal effect at 1-month follow-up, with AUC (mean cross-validation AUC, 0.80; 95% CI, 0.78-0.81), sensitivity (mean, 0.77; 95% CI, 0.75-0.78), specificity (mean, 0.78; 95% CI, 0.76-0.79), and positive predictive value (mean, 0.74; 95% CI, 0.72-0.77) further confirming the feasibility of using only EMA data.

### EMA Analysis

#### EMA Predictive Performance

As shown in Figure 1 and eFigures 2 and 3 in Supplement 1, the AUC was optimal when using all 25 days of EMA data at 1, 3, and 8 months’ follow-up, and the modeling approach consistently maintained acceptable accuracy (AUC >0.70) using approximately 11 days of EMA data (at 1 month, 10 days of data; at 3 months, 11 days; at 8 months, 12 days). Furthermore, mean AUCs obtained at 3-month follow-up over a 25-day time variation ranged from 0.53 to 0.80, 0.51 to 0.76, and 0.51 to 0.74.

Separate predictive performance evaluation was carried out for the 3 Chinese Lunar New Year waves at 3-month follow-up, and the results are shown in Figure 1 and eFigures 2 and 3 in Supplement 1 (eTable 3 in Supplement 1 has specific values). The best performance was obtained under wave 2 (during Lunar New Year) at 1-month follow-up, which had a mean AUC of 0.77 (95% CI, 0.74-0.79), a mean sensitivity of 0.74 (95% CI, 0.72-0.76), a mean specificity of 0.75 (95% CI, 0.73-0.77), and a mean positive predictive value of 0.72 (95% CI, 0.71-0.74). Likewise, the wave 2 metrics achieved consistent performance improvements over the other 2 waves at 3 and 8 months’ follow-up.

#### EMA Predictor Importance

**Figure 2.**
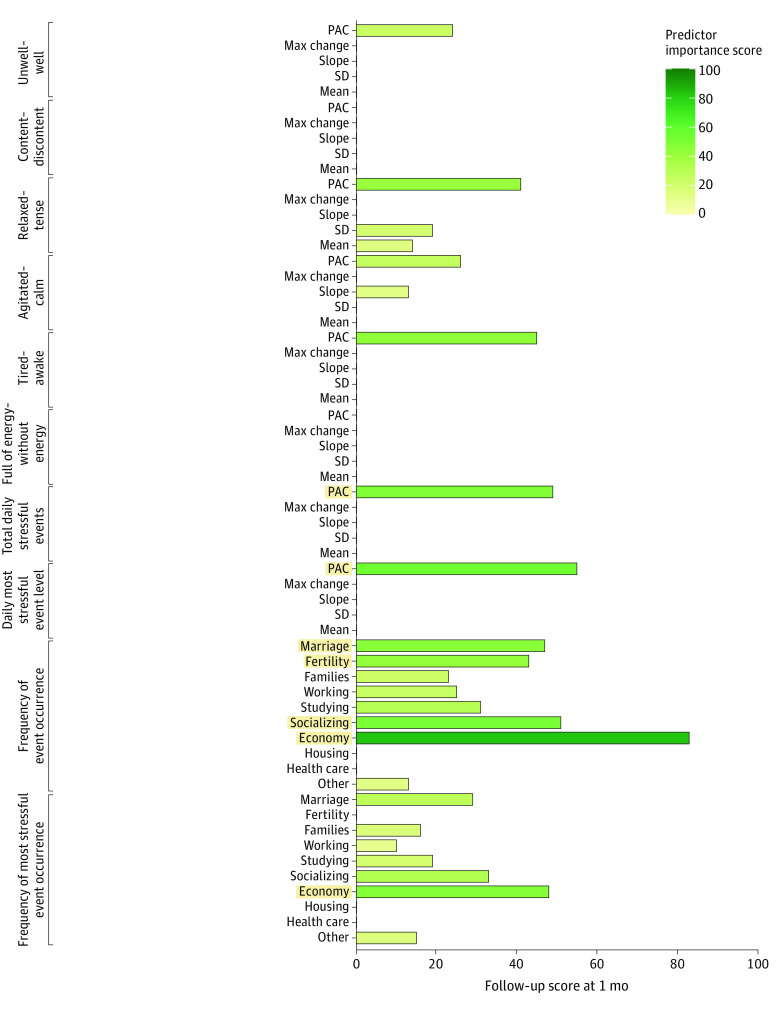
Measures of Feature Importance of Modeling Approach of Suicidal Ideation When Using All 25 Days of Ecological Momentary Assessment (EMA) Data at 1-Month Follow-Up PAC indicates probability of acute change.

**Figure 3.**
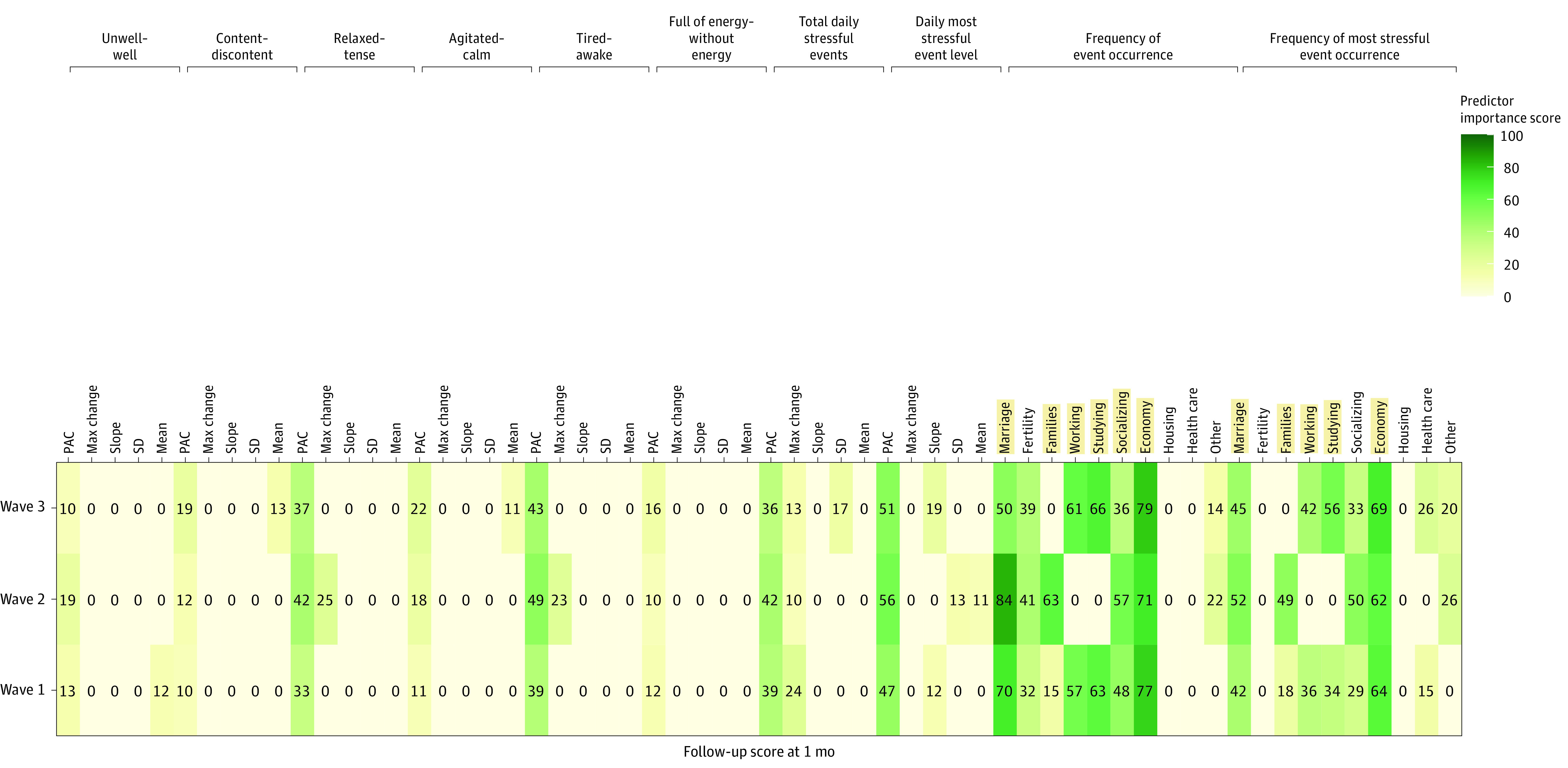
Measures of Feature Importance of Modeling Approach of Suicidal Ideation When Using Ecological Momentary Assessment (EMA) Data From 3 Different Chinese Lunar New Year Waves at 1-Month Follow-Up PAC indicates probability of acute change.

Modeling approach predictor importance scores (scale from 0 to 100, with 0 indicating least important and 100 indicating most important) are presented in Figures 2 and 3 and eFigures 4 and 5 in Supplement 1. When all 25 days of EMA data were used (Figure 2; eFigure 4 in Supplement 1), the most important EMA features at 1, 3, and 8 months’ follow-up were the frequency of marriage event (importance scores at 3-month follow-up: 47, 57, and 67), frequency of fertility event (43, 53, and 57), frequency of socializing event (51, 61, and 81), frequency of economy event (83, 93, and 97), frequency of most stressful economy event (48, 58, and 68), probability of acute change daily stressful event number (49, 56, and 54), and probability of acute change daily most stressful event level (55, 67, and 61).

Figure 3 and eFigure 5 in Supplement 1 present feature importance at 3-month follow-up from the 3 Chinese Lunar New Year waves. Features important for all 3 waves at 3-month follow-up were: frequency of marriage event, frequency of socializing event, frequency of economy event, frequency of most stressful marriage event, and frequency of most stressful economy event. The frequency of family event, frequency of working event, frequency of studying event, frequency of most stressful family event, frequency of most stressful working event, and frequency of most stressful studying event changed over the 3 waves. Refer to Figure 3 and eFigure 5 in Supplement 1 for specific importance scores for the 3 waves at 3-month follow-up.

#### Influence of Different Validation Procedures

The prediction performance metrics remained largely unchanged when different cross-validation procedures were used (eFigure 6 in Supplement 1). For the prediction at 1-month follow-up under 10-fold and leave-one-out cross-validation, the modeling approach performed best under wave 2 for almost all evaluation metrics (see eTables 4 and 5 in Supplement 1 for specific values).

## Discussion

To our knowledge, this study is the first to predict suicidal ideation among Chinese sexual and gender minority individuals that considered the interaction between mood fluctuations, stressful events, and the unique cultural context of Chinese Lunar New Year using intensive data and machine learning. This approach may contribute to early preventive interventions for this vulnerable population by highlighting the importance of prioritizing specific symptoms to reduce suicide risk during dynamic cultural phenomena.

The study demonstrates that EMA data had a stronger predictive effect for short-term suicidal ideation in sexual and gender minority individuals compared with baseline or a combination of baseline and EMA data. Additionally, our findings extend our previous study^27^ by demonstrating no significant difference in predictive effect between EMA and EMA plus baseline data. This suggests that EMA can be a valuable tool for identifying and addressing short- and long- term suicidal ideation in this population.^28^

Additionally, the approach consistently maintained acceptable accuracy using 11 to 14 days of EMA data. This finding supports the assertion that short-term risk factors, including daily emotional changes and stressful events, may be predictive of both imminent suicide and longer-term suicidal ideation.^29^ It could also be useful in cases where sexual and gender minority individuals have not self-reported suicidal ideation or have not yet experienced it, aiding in improved identification and treatment of those at risk of future suicidal ideation.^30^

Compared with other machine learning methods that use EMA data for predicting suicidal ideation, our approach demonstrated an acceptable level of accuracy. For instance, a study conducted by Horwitz et al^20^ achieved suicide ideation prediction with an AUC of 0.73 by incorporating emotional variables collected during an EMA period using elastic network regression. Nonetheless, when compared with studies in other application scenarios focusing on predicting outcomes related to suicide, our AUC value does not stand out. A study by Peis et al^31^ integrated health care records with EMA data extracted from a sample of 1023 participants and achieved an AUC of 0.83 using a recursive neural network. Studies conducted by Lekkas et al^32^ and Roy et al^33^ used ensemble learning and random forests in combination with social media data to forecast suicidal ideation, yielding AUC values ranging from 0.77 to 0.88. Future studies with larger sample sizes are necessary to replicate these findings and determine whether modeling approaches incorporating EMA data can achieve satisfactory AUC scores.

The wave during Chinese Lunar New Year showed the best prediction for later suicidal ideation in sexual and gender minority individuals compared with before and after Chinese Lunar New Year. This finding is consistent with the so-called Chinese New Year effect, which reveals a higher mortality risk among patients admitted to the hospital during the holiday.^34^ Another possible explanation specific to the population in our study is that the Chinese Lunar New Year holds immense cultural significance as the most important event for family reunions. Intensive meetings with many family members over several weeks may trigger more daily stressful events with emotional fluctuations, such as discussions about marriage and children.^35,36^ The traditional norm of filial piety,^37^ which strongly restricts communication with parents or other family members regarding their minority sexual identity, may amplify the negative emotions caused by denial or conflict.^38,39,40^ More interestingly, a previous study claims that the suicide rate among Chinese elderly individuals decreased by 8.7% during Chinese Lunar New Year, a time when they received an unusually high level of family companionship. However, no similar effects were found among younger and middle-aged cohorts.^41^ These findings further emphasize another side of the coin of the happy family gathering time (eg, Chinese Lunar New Year) for some sexual and gender minority individuals.^42^ This highlights the need for further exploration to understand the underlying mechanisms and pathways that operate within this special cultural context.

Furthermore, family-related stressors were found to be a prominent predictive factor for suicidal ideation during Chinese Lunar New Year, whereas academic and work-related factors were only significant predictors before and after Lunar New Year. This finding provides indirect evidence that sexual and gender minority individuals may experience heightened social and familial stressors during this period, particularly related to topics such as children, marriage, or finances.^12,37,38^ These obligations could potentially distract them from academic and work-related stressors.

The study found that emotional changes over a short period were important predictors of short-term suicidal ideation, particularly changes between relaxed-tense and tired-awake states; however, this influence weakened over time. Consistent with the mood-as-information theory,^43^ which suggests that individuals often rely on their current emotional state as a source of information when making judgments or decisions, sexual and gender minority individuals may tend to overinterpret the negative aspects of their current situation when transitioning from a relaxed or awake state to a tense or tired state. This in turn may lead to increased suicidal ideation in the short term.^44^ However, individuals may begin to reevaluate and distance themselves from their current mood states, weakening the association between mood changes and suicidal ideation.^45^

### Limitations

The results of this study must be interpreted in light of its limitations. First, the self-report measurements used in the study may have yielded biased estimates. To confirm the validity of current results, multiple measures were used to triangulate findings.^46^ Additionally, we only used a single item to assess suicidal ideation, although the effectiveness has been proven in previous studies,^19,20,21^ more comprehensive assessments of suicidal ideation and behaviors are required. Our study used daily fixed-time prompts, and the introduction of daily random-time assessments will further enhance the ecological validity. Additionally, the inclusion of more relevant items in each survey to increase the number of variables will enable meaningful predictions of suicidal ideation in the morning of the following day based on the data from the previous day. We acknowledge that the effectiveness of the modeling approach has not been fully substantiated through cross-validation techniques and is still undergoing internal validation. It is preferable to introduce additional independent data sets for external validation. Suicide attempt was also measured but due to a limited sample size it was not possible to develop a machine learning approach for this outcome. Meanwhile, more empirical studies are calling to focus specifically on Chinese holidays and their possible effect. Moreover, it is worth considering cost-effectiveness analysis in future studies as an effective strategy to assist decision-making regarding the value of these tools in real-world settings.

## Conclusions

This diagnostic study found optimal predictive effects for 1 month of suicidal ideation in sexual and gender minority individuals. This was achieved by using a machine learning approach that incorporated mood fluctuations and stressful events as predictors. The role of emotion weakened over time, while stressors related to work, finances, fidelity, and marriage were still strong in predicting suicidal ideation at 3 and 8 months. The modeling approach developed in this study revealed distinct underlying mechanisms in the development of short-term and longer-term suicidal ideation. These findings provide insight into the potential creation of an identification tool to complement early prevention efforts targeting suicide risk in this population.

## Notes

Supplement 1.
eMethods.

eReferences
**eTable 1.** Hyperparameter space search for LightGBM **eTable 2.** Modeling approach performance metrics using baseline, EMA, and baseline+EMA data to predict suicidal ideation at 1, 3, and 8 months of follow-up **eTable 3.** Modeling approach performance metrics using EMA data to predict suicidal ideation under three different waves at 1, 3, and 8 months of follow-up **eTable 4.** Modeling approach performance metrics using EMA data to predict suicidal ideation by 10-fold cross-validation under three different waves at 1 month follow-up **eTable 5.** Modeling approach performance metrics using EMA data to predict suicidal ideation by leave-one-out cross-validation under three different waves at 1 month follow-up **eFigure 1.** The flowchart indicating exclusions for primary analyses and the different steps of the study **eFigure 2.** Performance of modeling approach in predicting suicidal ideation at 3 months follow-up **eFigure 3.** Performance of modeling approach in predicting suicidal ideation at 8 months follow-up **eFigure 4.** Measures of feature importance of modeling approach of suicidal ideation when using all 25 days EMA data at 3 and 8 months of follow-up **eFigure 5.** Measures of feature importance of modeling approach of suicidal ideation when using EMA data from three different Chinese Lunar New Year waves at 3 and 8 months of follow-up **eFigure 6.** Validation of the different validation procedures at 1 month follow-up

Supplement 2.
Data Sharing Statement
