# Supplementary material for: Ecological Momentary Assessment and Machine Learning for Predicting Suicidal Ideation Among Sexual and Gender Minority Individuals
Source: JAMA Netw Open. 2023 Sep 11;6(9):e2333164. doi: 10.1001/jamanetworkopen.2023.33164 (PMC10495869; doi:10.1001/jamanetworkopen.2023.33164)
Supplement: Supplement 1. — eMethods. eReferences eTable 1. Hyperparameter space search for LightGBM eTable 2. Modeling approach performance metrics using baseline, EMA, and baseline+EMA data to predict suicidal ideation at 1, 3, and 8 months of follow-up eTable 3. Modeling approach performance metrics using EMA data to predict suicidal ideation under three different waves at 1, 3, and 8 months of follow-up eTable 4. Modeling approach performance metrics using EMA data to predict suicidal ideation by 10-fold cross-validation under three different waves at 1 month follow-up eTable 5. Modeling approach performance metrics using EMA data to predict suicidal ideation by leave-one-out cross-validation under three different waves at 1 month follow-up eFigure 1. The flowchart indicating exclusions for primary analyses and the different steps of the study eFigure 2. Performance of modeling approach in predicting suicidal ideation at 3 months follow-up eFigure 3. Performance of modeling approach in predicting suicidal ideation at 8 months follow-up eFigure 4. Measures of feature importance of modeling approach of suicidal ideation when using all 25 days EMA data at 3 and 8 months of follow-up eFigure 5. Measures of feature importance of modeling approach of suicidal ideation when using EMA data from three different Chinese Lunar New Year waves at 3 and 8 months of follow-up eFigure 6. Validation of the different validation procedures at 1 month follow-up [file jamanetwopen-e2333164-s001.pdf]

Supplemental Online Content

Lei C, Qu D, Liu K, Chen R. Ecological momentary assessment and machine learning for predicting suicidal ideation among sexual and gender minority individuals. *JAMA Netw Open*. 2023;6(9):e2333164.
doi:10.1001/jamanetworkopen.2023.33164

eMethods

eReferences

eTable 1. Hyperparameter space search for LightGBM

eTable 2. Modeling approach performance metrics using baseline, EMA, and baseline+EMA data to predict suicidal ideation at 1, 3, and 8 months of follow-up

eTable 3. Modeling approach performance metrics using EMA data to predict suicidal ideation under three different waves at 1, 3, and 8 months of follow-up

eTable 4. Modeling approach performance metrics using EMA data to predict suicidal ideation by 10-fold cross-validation under three different waves at 1 month follow-up

eTable 5. Modeling approach performance metrics using EMA data to predict suicidal ideation by leave-one-out cross-validation under three different waves at 1 month follow-up

eFigure 1. The flowchart indicating exclusions for primary analyses and the different steps of the study

eFigure 2. Performance of modeling approach in predicting suicidal ideation at 3 months follow-up

eFigure 3. Performance of modeling approach in predicting suicidal ideation at 8 months follow-up

eFigure 4. Measures of feature importance of modeling approach of suicidal ideation when using all 25 days EMA data at 3 and 8 months of follow-up

© 2023 Lei C et al. *JAMA Network Open.*

eFigure 5. Measures of feature importance of modeling approach of suicidal ideation when using EMA data from three different Chinese Lunar New Year waves at 3 and 8 months of follow-up

eFigure 6. Validation of the different validation procedures at 1 month follow-up

This supplemental material has been provided by the authors to give readers additional information about their work.

eMethods

Machine learning modeling approach

The light gradient boosting machine (LightGBM) is a lightweight algorithm based on the gradient boosting decision tree (GBDT) algorithm developed by Microsoft. The GBDT algorithm is an iterative based decision tree algorithm. LightGBM uses a histogram-based algorithm (Histogram) to traverse each segmentation point, which consumes less memory and reduces the complexity of data separation to speed up the training process. It also uses the exclusive features bundling algorithm to bind many mutually exclusive features into one feature, and uses the leaf-wise strategy to grow trees and find the leaf with the largest gain of variance for the split. LightGBM thus has the advantages of faster training speed, lower memory usage, and higher accuracy.

Hyperparameters tuning of modeling approach

Hyperparameters are adjustable parameters that control the model training process and dramatically influence the performance of the model. To identify the optimal set of parameters, we employed a 5-fold cross-validation strategy. This involved iteratively training the model using 1,000 different sets of hyperparameters. The selection of the best parameter set was based on evaluating the AUC metric. The range of hyperparameter search and the specific values ultimately employed are presented in eTable 1.

Predictors importance ranking

To mitigate potential bias stemming from the utilization of a single set of hyperparameters, we trained 1,000 models across diverse parameter spaces. Subsequently, we selected the top 5% (50 out of 1000) models based on their AUCs. The final importance score ranking was calculated by averaging those best-performed models and sorted.

Cross-validation evaluation

© 2023 Lei C et al. *JAMA Network Open.*

The five-fold cross-validation scheme was applied and repeated three times. The original data sets were randomly divided into five equal sized subsets. Of the five subsets, a single subset was retained as the validation data to test the predictive ability of the model, whereas the remaining four subsets were used as training samples to estimate model parameters. The results were presented across the folds by calculating the average statistics along with a 95% confidence interval.

© 2023 Lei C et al. *JAMA Network Open.*

eReferences

1. Ke G, Meng Q, Finley T, et al. Lightgbm: a highly efficient gradient boosting decision tree. *Adv. Neural Inf. Process. Syst.* 2017; 30. doi:10.1046/j.1365-2575.1999.00060.x

2. Pedregosa F, Varoquaux G, Gramfort A, et al. Scikit-learn: machine learning in Python. *J. Mach. Learn. Res.* 2011; 12(Oct): 2825-2830.

3. Zhou Z H. *Ensemble Methods: Foundations and Algorithms*. 2012. CRC press.

4. Kuhn M, Johnson K. *Applied Predictive Modeling*. Springer-Verlag; 2013. doi:10.1007/978-1-4614-6849-3

5. Kuhn M, Johnson K, Kuhn M, et al. Over-fitting and model tuning. *Appl. Predict. Model.* 2013; 61-92. doi:10.1007/978-1-4614-6849-3

© 2023 Lei C et al. *JAMA Network Open.*

eTable 1. Hyperparameter space search for LightGBM

Hyperparameters	Search range	Step	Final adoption
n_estimators	[100, …, 1000]	100	500
num_leaves	[10, …, 100]	10	10
max_depth	[3, …, 30]	3	15
subsample	[0.7, …, 1]	0.05	0.7
colsample_bytree	[0.7, …, 1]	0.05	0.7
learning_rate	[1e-5, …, 1e-1]	*10	1e-2

© 2023 Lei C et al. *JAMA Network Open.*

eTable 2. Modeling approach performance metrics using baseline, EMA, and baseline+EMA data to predict suicidal ideation at 1, 3, and 8 months of follow-up

	AUC Mean[95%CI]	SEN Mean[95%CI]	SPE Mean[95%CI]	PPV Mean[95%CI]
**Baseline**				
1 month	0.635 [0.613-0.657]	0.574 [0.552-0.597]	0.594 [0.572-0.617]	0.536 [0.516-0.556]
3 months	0.625 [0.609-0.642]	0.576 [0.559-0.593]	0.595 [0.578-0.613]	0.525 [0.511-0.540]
8 months	0.623 [0.608-0.638]	0.583 [0.567-0.599]	0.602 [0.587-0.616]	0.524 [0.510-0.539]
**EMA**				
1 month	0.797 [0.780-0.814]	0.766 [0.748-0.784]	0.777 [0.761-0.793]	0.743 [0.718-0.767]
3 months	0.764 [0.748-0.781]	0.733 [0.706-0.760]	0.743 [0.720-0.765]	0.703 [0.687-0.719]
8 months	0.744 [0.729-0.759]	0.713 [0.697-0.729]	0.724 [0.710-0.738]	0.685 [0.671-0.698]
**Baseline+EMA**				
1 month	0.763 [0.749-0.776]	0.729 [0.717-0.741]	0.741 [0.732-0.750]	0.697 [0.685-0.708]
3 months	0.763 [0.747-0.779]	0.731 [0.710-0.753]	0.744 [0.718-0.769]	0.701 [0.690-0.713]
8 months	0.745 [0.733-0.757]	0.713 [0.697-0.729]	0.724 [0.709-0.739]	0.684 [0.670-0.698]

Results were calculated based on 5-fold cross-validation with 3 repetitions.
Abbreviations: AUC = Area under the receiver operating characteristic (ROC) curve, SEN = TP/TP+FN, SPE = TN/TN+FP, PPV = TP/TP+FP, TP = True positive, TN = True negative, FP = False positive, FN = False negative.

© 2023 Lei C et al. *JAMA Network Open.*

eTable 3. Modeling approach performance metrics using EMA data to predict suicidal ideation under three different waves at 1, 3, and 8 months of follow-up

	AUC Mean[95%CI]	SEN Mean[95%CI]	SPE Mean[95%CI]	PPV Mean[95%CI]
**wave 1**				
1 month	0.682 [0.660-0.704]	0.665 [0.643-0.687]	0.675 [0.657-0.693]	0.641 [0.627-0.654]
3 months	0.647 [0.616-0.678]	0.634 [0.609-0.658]	0.643 [0.623-0.664]	0.609 [0.593-0.626]
8 months	0.625 [0.593-0.658]	0.612 [0.586-0.638]	0.622 [0.599-0.644]	0.588 [0.570-0.606]
**wave 2**				
1 month	0.768 [0.742-0.794]	0.742 [0.721-0.763]	0.748 [0.730-0.766]	0.720 [0.705-0.735]
3 months	0.737 [0.715-0.759]	0.711 [0.693-0.728]	0.717 [0.703-0.731]	0.689 [0.677-0.700]
8 months	0.715 [0.693-0.737]	0.689 [0.672-0.706]	0.695 [0.681-0.709]	0.667 [0.655-0.679]
**wave 3**				
1 month	0.664 [0.644-0.684]	0.644 [0.624-0.665]	0.637 [0.613-0.661]	0.613 [0.594-0.632]
3 months	0.633 [0.610-0.655]	0.613 [0.591-0.635]	0.606 [0.580-0.632]	0.581 [0.560-0.603]
8 months	0.611 [0.590-0.632]	0.591 [0.571-0.611]	0.584 [0.560-0.609]	0.560 [0.540-0.580]

Results were calculated based on 5-fold cross-validation with 3 repetitions.
Abbreviations: AUC = Area under the receiver operating characteristic (ROC) curve, SEN = TP/TP+FN, SPE = TN/TN+FP, PPV = TP/TP+FP, TP = True positive, TN = True negative, FP = False positive, FN = False negative.

eTable 4. Modeling approach performance metrics using EMA data to predict suicidal ideation by 10-fold cross-validation under three different waves at 1 month follow-up

	**AUC** Mean[95%CI]	**SEN** Mean[95%CI]	**SPE** Mean[95%CI]	**PPV** Mean[95%CI]
**1 month**				
wave 1	0.686 [0.664-0.707]	0.674 [0.654-0.694]	0.655 [0.635-0.676]	0.625 [0.607-0.643]
wave 2	0.761 [0.733-0.788]	0.752 [0.730-0.774]	0.728 [0.707-0.749]	0.701 [0.684-0.717]
wave 3	0.658 [0.638-0.677]	0.655 [0.635-0.674]	0.620 [0.596-0.643]	0.594 [0.572-0.616]

Results were calculated based on 10-fold cross-validation with 3 repetitions.
Abbreviations: AUC = Area under the receiver operating characteristic (ROC) curve, SEN = TP/TP+FN, SPE = TN/TN+FP, PPV = TP/TP+FP, TP = True positive, TN = True negative, FP = False positive, FN = False negative.

© 2023 Lei C et al. *JAMA Network Open.*

eTable 5. Modeling approach performance metrics using EMA data to predict suicidal ideation by leave-one-out cross-validation under three different waves at 1 month follow-up

	AUC Mean[95%CI]	SEN Mean[95%CI]	SPE Mean[95%CI]	PPV Mean[95%CI]
**1 month**				
wave 1	0.668 [0.644-0.692]	0.670 [0.648-0.692]	0.663 [0.641-0.685]	0.621 [0.601-0.641]
wave 2	0.740 [0.713-0.767]	0.749 [0.731-0.767]	0.735 [0.717-0.753]	0.698 [0.682-0.714]
wave 3	0.639 [0.619-0.659]	0.652 [0.632-0.672]	0.625 [0.601-0.649]	0.591 [0.567-0.615]

Results were calculated based on leave-one-out cross-validation.
Abbreviations: AUC = Area under the receiver operating characteristic (ROC) curve, SEN = TP/TP+FN, SPE = TN/TN+FP, PPV = TP/TP+FP, TP = True positive, TN = True negative, FP = False positive, FN = False negative.

© 2023 Lei C et al. *JAMA Network Open.*

eFigure 1. Flowchart indicating exclusions for primary analyses and the different steps of the study

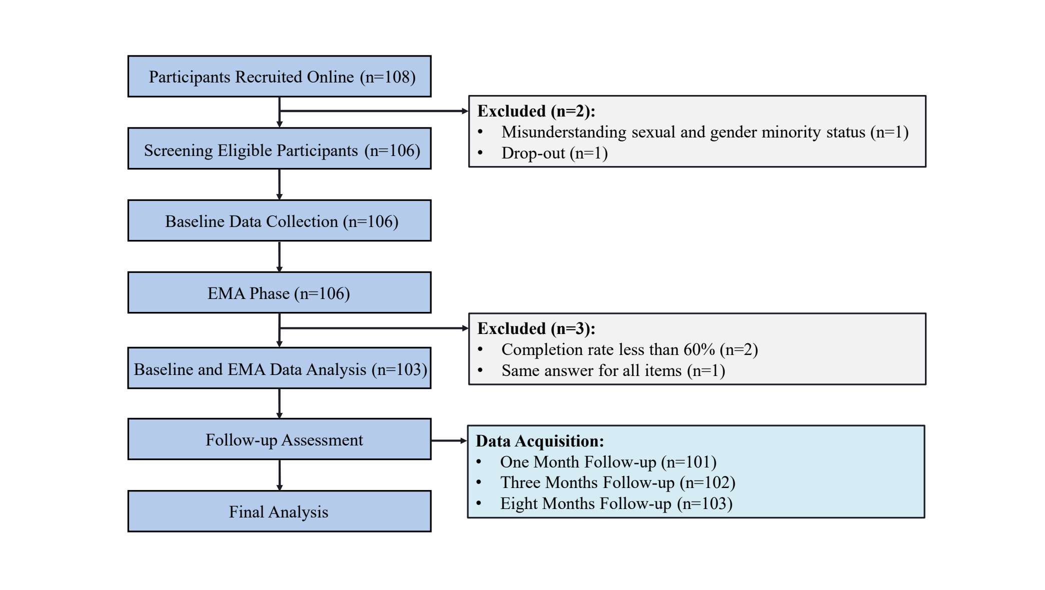

© 2023 Lei C et al. *JAMA Network Open.*

eFigure 2. Performance of modeling approach in predicting suicidal ideation at 3 months follow-up

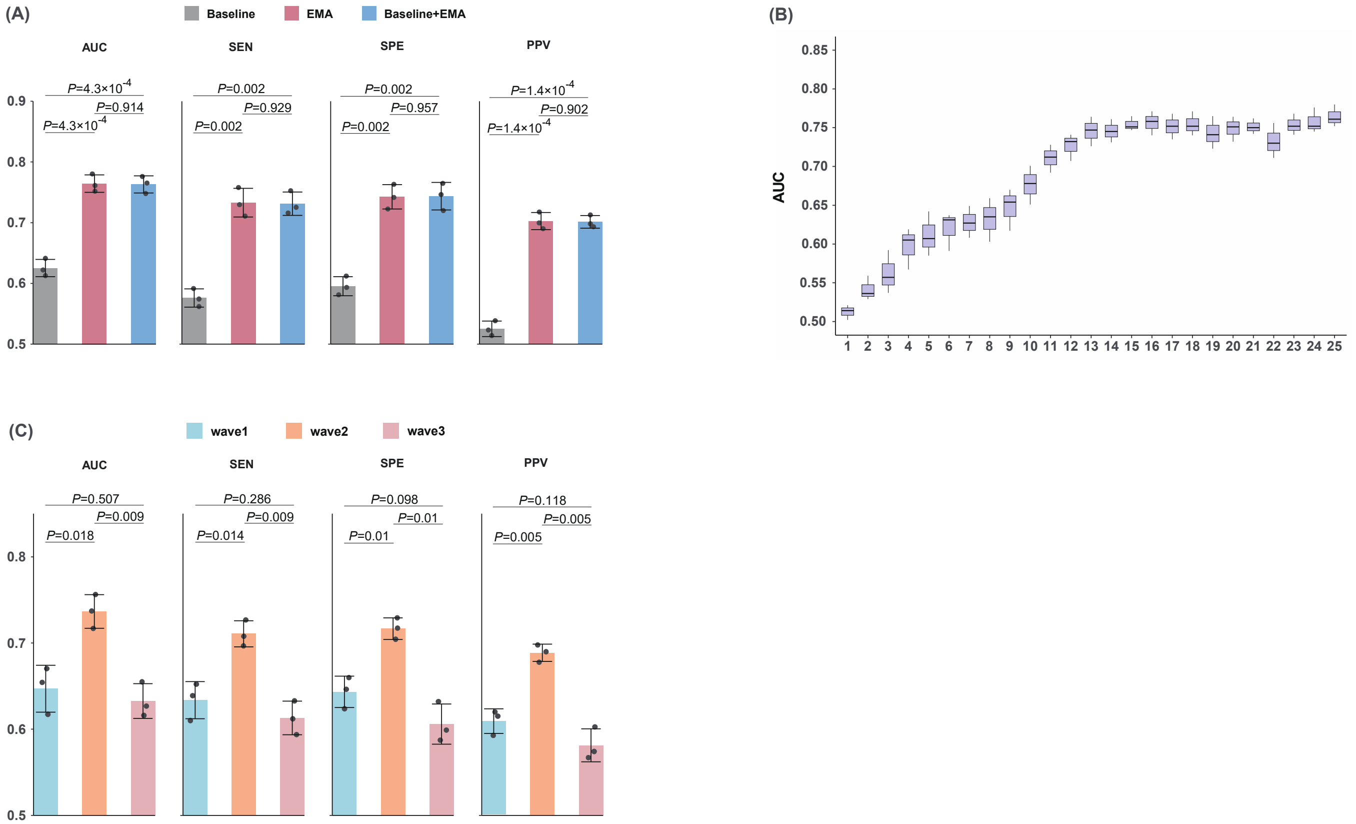

(A) Comparison of predictive performance of using baseline, EMA, and baseline+EMA data; (B) Changes in the AUC when the daily EMA data are continually overlaid to predict; (C) Evaluation of predictive performance of three different waves. Differences among two groups were calculated with *t*-tests: *P* values were adjusted for multiple comparisons by using false discovery rate (FDR) adjustment (Benjamini-Hochberg procedure).

© 2023 Lei C et al. *JAMA Network Open.*

eFigure 3. Performance of modeling approach in predicting suicidal ideation at 8 months follow-up

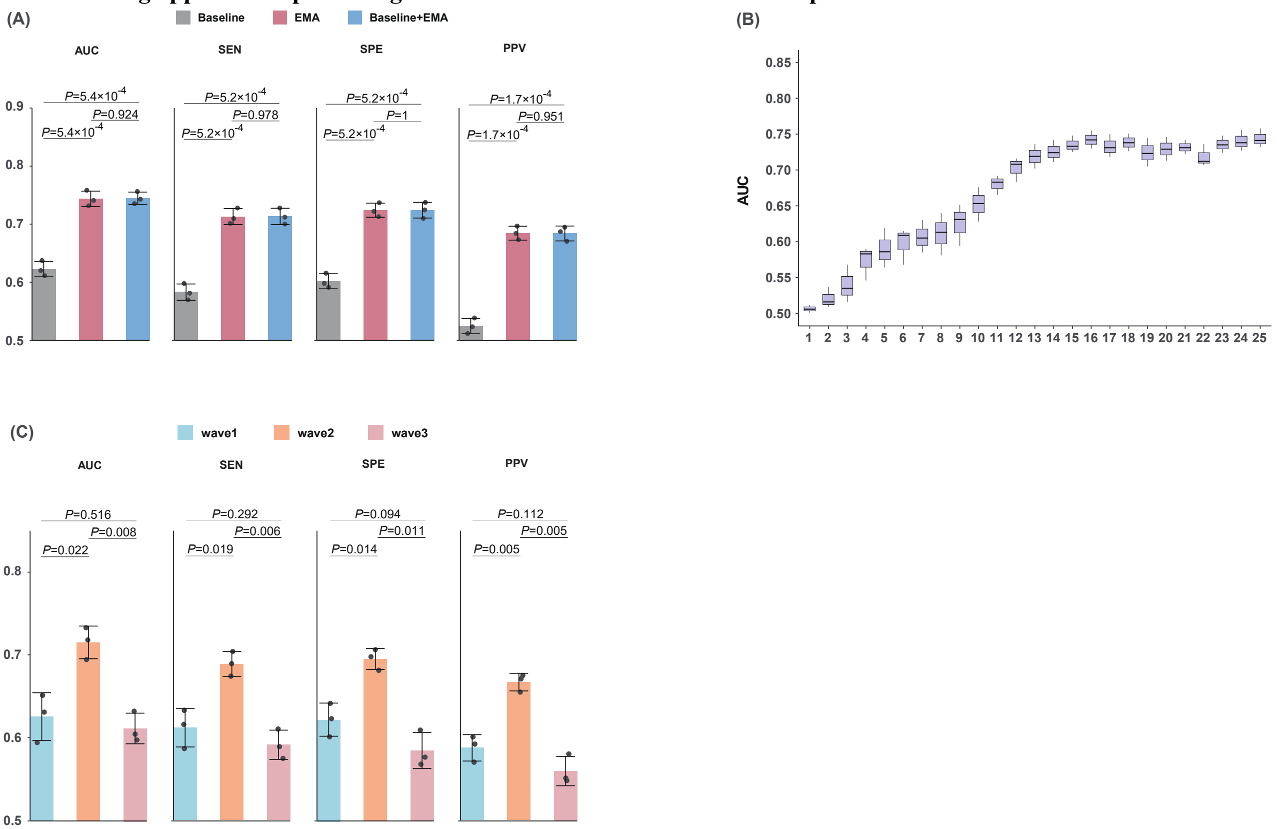

(A) Comparison of predictive performance of using baseline, EMA, and baseline+EMA data; (B) Changes in the AUC when the daily EMA data are continually overlaid to predict; (C) Evaluation of predictive performance of three different waves. Differences among two groups were calculated with *t*-tests: *P* values were adjusted for multiple comparisons by using false discovery rate (FDR) adjustment (Benjamini-Hochberg procedure).

© 2023 Lei C et al. *JAMA Network Open.*

eFigure 4. Measures of feature importance of modeling approach of suicidal ideation when using all 25 days EMA data at 3 and 8 months of follow-up

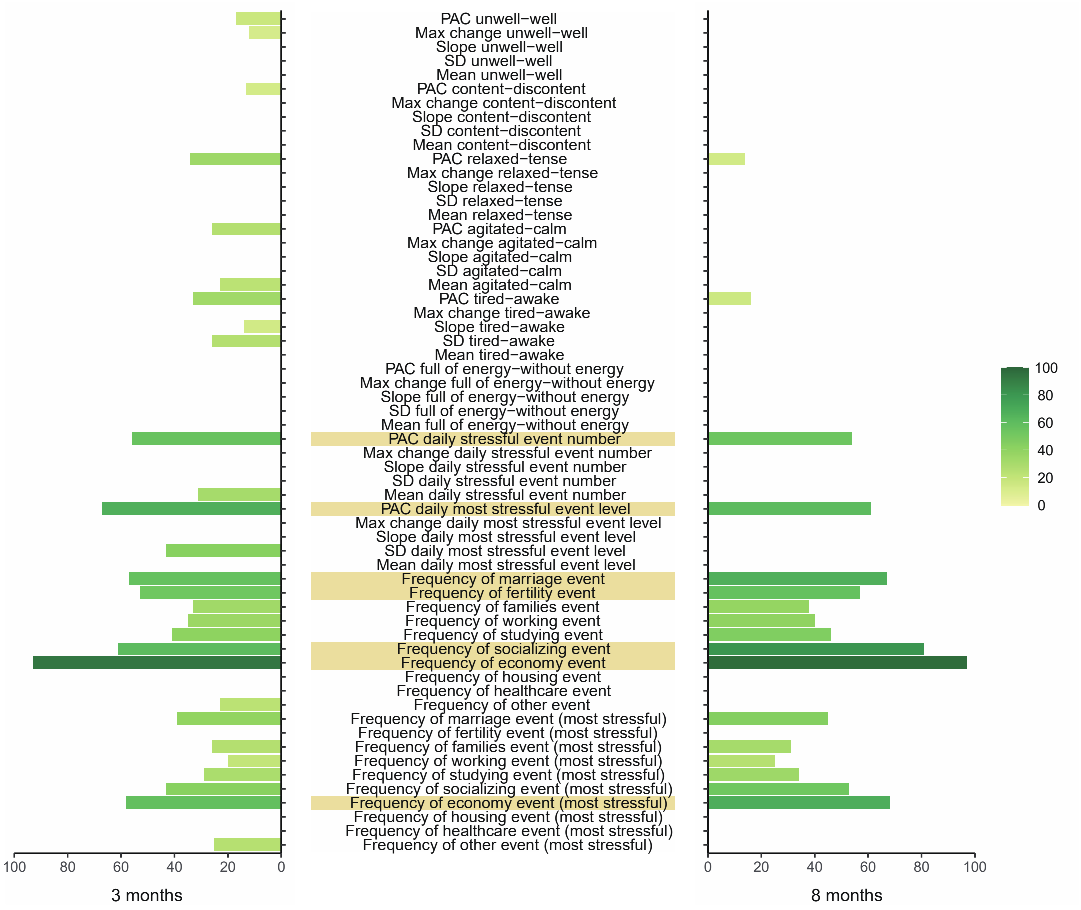

© 2023 Lei C et al. *JAMA Network Open.*

eFigure 5. Measures of feature importance of modeling approach of suicidal ideation when using EMA data from three different Chinese Lunar New Year waves at 3 and 8 months of follow-up

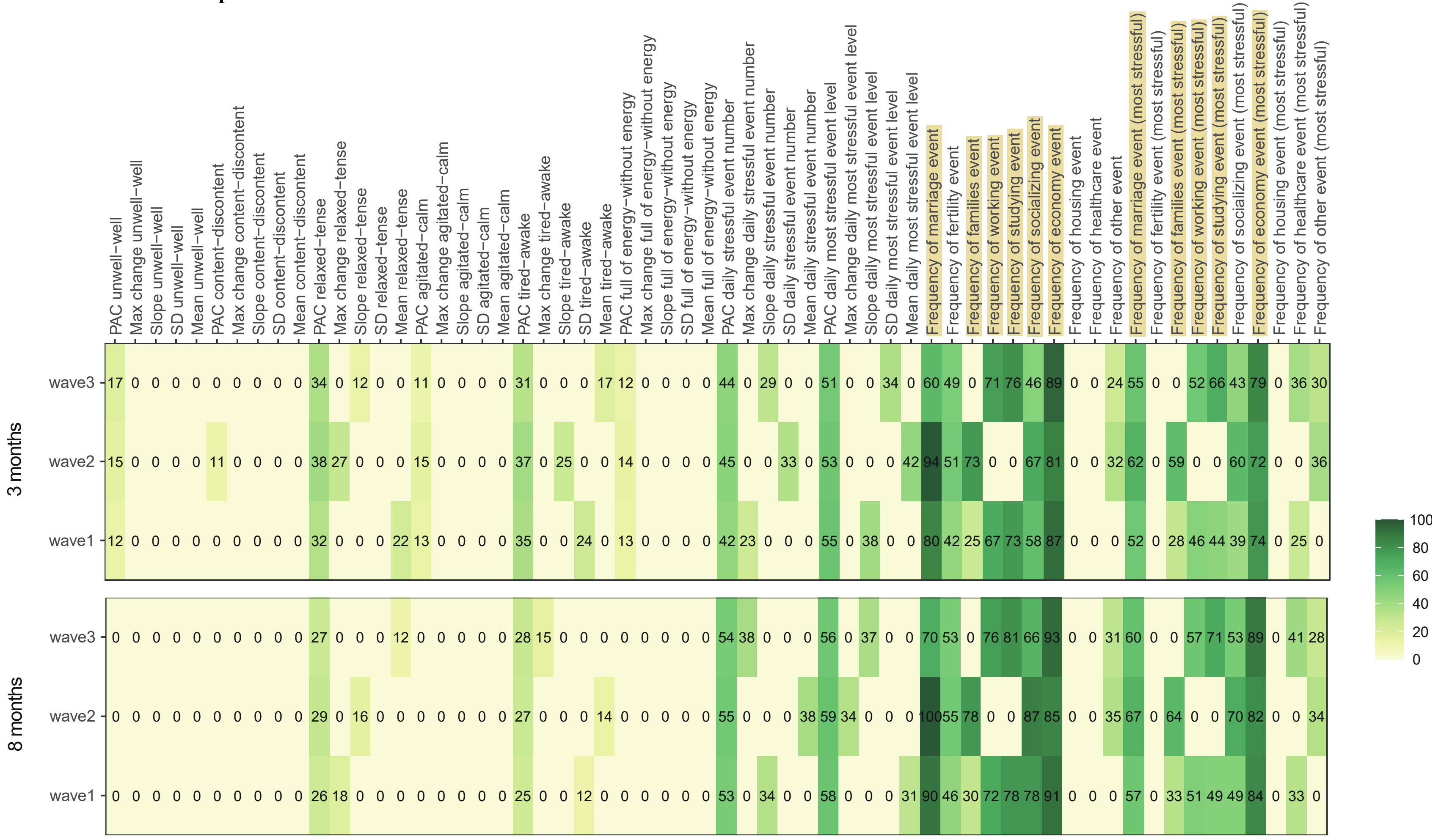

© 2023 Lei C et al. *JAMA Network Open.*

eFigure 6. Validation of the different validation procedures at 1 month follow-up

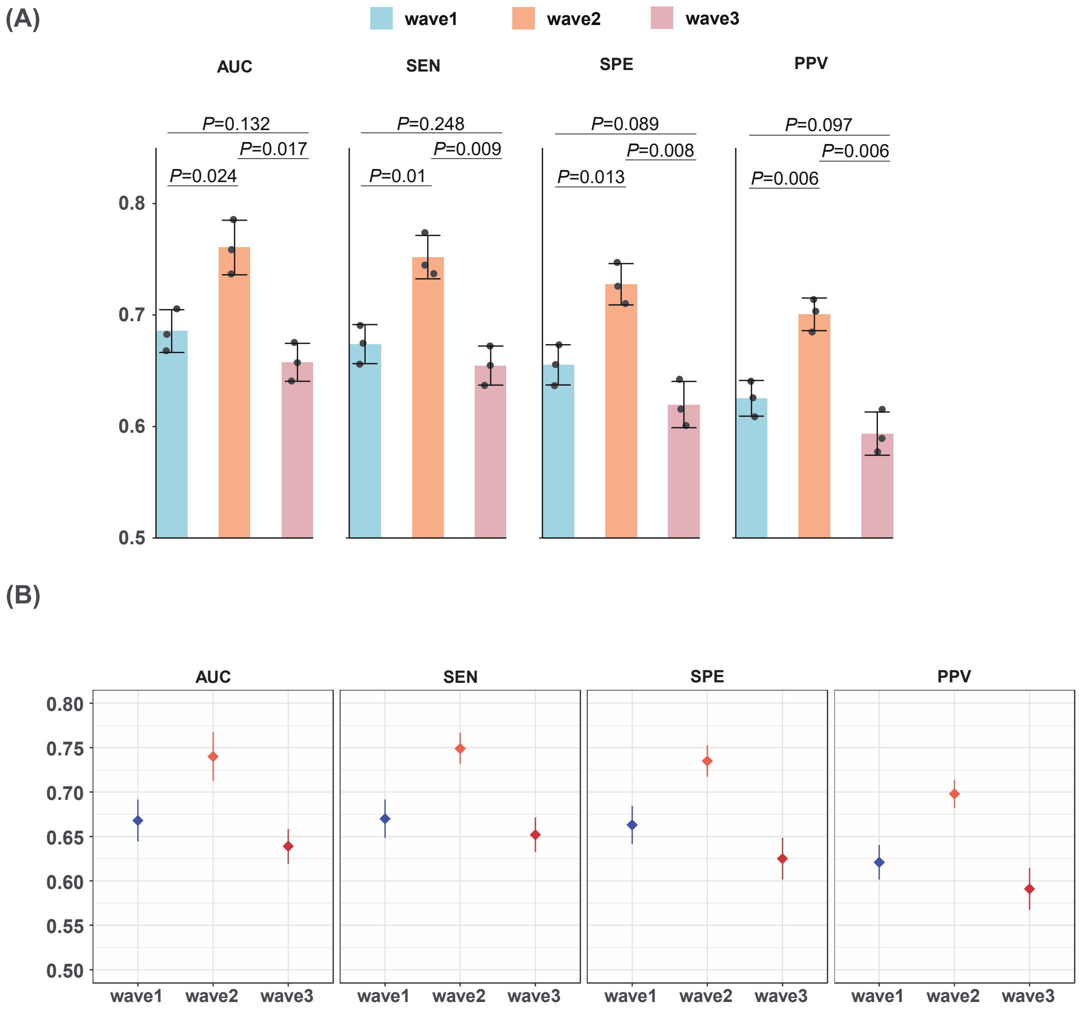

(A) Modeling approach performance metrics with 10-fold cross-validation repeated 3 times; (B) Modeling approach performance metrics for leave-one-out cross-validation (the average values with 95% CI). Differences among two groups were calculated with *t*-tests: *P* values were adjusted for multiple comparisons by using false discovery rate (FDR) adjustment (Benjamini-Hochberg procedure).

© 2023 Lei C et al. *JAMA Network Open.*
