# Supplementary material for: Ecological Momentary Assessment and Machine Learning for Predicting Suicidal Ideation Among Sexual and Gender Minority Individuals
Source: JAMA Netw Open. 2023 Sep 11;6(9):e2333164. doi: 10.1001/jamanetworkopen.2023.33164 (PMC10495869; doi:10.1001/jamanetworkopen.2023.33164)
Supplement: Supplement 2. — Data Sharing Statement [file jamanetwopen-e2333164-s002.pdf]

Data Sharing Statement

Lei. Ecological Momentary Assessment and Machine Learning for Predicting Suicidal Ideation Among Sexual and Gender Minority Individuals. *JAMA Netw Open*. Published September 11, 2023. doi:10.1001/jamanetworkopen.2023.33164

Data
Data available: No

Additional Information
Explanation for why data not available: Data are not publicly available due to their containing information that could compromise research participant privacy/consent. We cannot make the data available to others or deposit it in public database without further ethical approval.
